# Inner Speech is not so Simple: A Commentary on Cho and Wu (2013)

**DOI:** 10.3389/fpsyt.2014.00042

**Published:** 2014-04-22

**Authors:** Peter Moseley, Sam Wilkinson

**Affiliations:** ^1^Department of Psychology, Durham University, Durham, UK; ^2^Department of Philosophy, Durham University, Durham, UK

**Keywords:** auditory hallucinations, explanation in psychology, self-monitoring, spontaneous activation, inner speech

We welcome Cho and Wu’s (1) suggestion that the study of auditory verbal hallucinations (AVHs) could be improved by contrasting and testing more explanatory models. However, we have some worries both about their criticisms of inner speech-based self-monitoring (ISS) models and whether their proposed spontaneous activation (SA) model is explanatory.

Cho and Wu rightly point out that some phenomenological aspects of inner speech do not seem concordant with phenomenological aspects of AVH; Langdon et al. (2) found that, while many AVHs took the third person form (“he/she”), this was a relatively rare occurrence in inner speech, both for patients with a diagnosis of schizophrenia who experienced AVHs and control participants. This is indeed somewhat problematic for ISS models, notwithstanding potential problems with the introspective measures used in the above study. However, Cho and Wu go on to ask: “how does inner speech in one’s own voice with its characteristic features become an AVH of, for example, the neighbor’s voice with its characteristic features?” (p. 2). Here, it seems that Cho and Wu simply assume that inner speech is always experienced in one’s own voice, and are not aware of research suggesting that the presence of other people’s voices *is exactly the kind of quality reported in typical inner speech*. For example, McCarthy-Jones and Fernyhough (3) showed that it is common for healthy, non-clinical participants to report hearing other voices as part of their inner speech, as well as to report their inner speech taking on the qualities of a dialogic exchange. This is consistent with Vygotskian explanations of the internalization of external dialogs during psychological development (4). In this light, no “transformation” from one’s own voice to that of another is needed, and no “additional mechanism” needs to be added to the ISS model (5).

In any case, this talk of transformation is misleading. There is no *experience* of inner speech *first*, which is then somehow transformed. The question about whether inner speech is implicated in AVHs is about whether elements involved in the production of inner speech experiences are also involved in the production of some AVHs. There seems to be fairly strong evidence to support this.

That inner speech involves motoric elements has been empirically supported by several electromyographical (EMG) studies [e.g., Ref. (6)]. Later experiments made the connection between inner speech and AVH, showing that similar muscular activation is involved in AVH (7, 8). The involvement of inner speech in AVH is further supported by the findings from Gould (9), who showed that when his subjects hallucinated, subvocalizations occurred which could be picked up with a throat microphone. These subvocalizations were causally responsible for the AVHs, and not just echoing them (as has been hypothesized to happen in some cases of verbal comprehension [cf. e.g., Ref. (10)]) was suggested by Bick and Kinsbourne (11), who demonstrated that if people experiencing hallucinations opened their mouths wide, stopping vocalizations, then the majority of AVHs stopped.

Cho and Wu argue that ISS models are no better than SA models at explaining the specificity of AVHs to specific voices and content; we would argue that an ISS model, with recognition that inner speech is more complex than one’s own voice speaking in the first person, explains more than the SA model, because it explains why voices with a specific phenomenology are experienced in the first place, as opposed to more random auditory experiences that might be expected from SA in auditory cortex. The appeal to individual differences in gamma synchrony as an underlying mechanism of SA also does not seem capable of explaining why this would lead to activations of specific voice representations.

Cho and Wu go on to say that “once we allow that a given episode of AVH involves the features of another person’s voice with its characteristic acoustic features, it is simple to explain why the patient misattributes the event to another person: that is what it sounds like” (p. 2). Taken to its extreme, this implies that any episode of inner speech that involves a voice other than one’s own would be experienced as “non-self”, and hence experienced as similar to an AVH, a proposition that would clearly not find much support in empirical research. Taking this view, it is the SA model that needs an additional mechanism to explain why neuronal representations of other people’s voices are experienced not just as *sounding* like someone else’s voice, but also having the *non-self-generated, alien* quality associated with AVHs. This is exactly the type of mechanism built into ISS models of AVHs.

Indeed, the authors do go on to argue that many problems with the inner speech model of AVHs can be solved if we stop referring to “inner speech”, and instead refer to “auditory imagination”, which, supposedly, is characterized by actual acoustical properties, unlike inner speech (the authors do not cite any literature to support this claim). We would argue that this falls within the realm of typical inner speech, and that the view put forward by Cho and Wu is based on unexamined assumptions about the typical form of inner speech. We would argue that a separate “type” of imagery is not needed, and it is probable that inner speech recruits at least some mechanisms of auditory imagery. Therefore, it does not make sense to argue that AVHs resemble one, but not the other.

Finally, it should be pointed out that auditory cortical regions are not the only areas reported to lead to AVHs when directly stimulated; for example, Bancaud et al. (12) reported that stimulating the anterior cingulate cortex (ACC), an area often associated with error monitoring and cognitive control, caused auditory hallucinations, a finding that seems more compatible with self-monitoring accounts of AVH. Admittedly, it is possible that stimulation of ACC could have distal effects, also stimulating auditory cortical regions; we mention this finding simply to highlight the fact that the potential top–down effects of other brain regions on auditory cortical areas should not be overlooked.

## Conflict of Interest Statement

The authors declare that the research was conducted in the absence of any commercial or financial relationships that could be construed as a potential conflict of interest.
